# Atypical *CTSK* Transcripts and *ARNT* Transcription Read-Through Into *CTSK*

**DOI:** 10.1002/cfg.483

**Published:** 2005

**Authors:** Fabienne S. Giraudeau, Jean-Philippe Walhin, Paul R. Murdock, Nigel K. Spurr, Ian C. Gray

**Affiliations:** 1 Department of Discovery Genetics GlaxoSmithKline Research and Development New Frontiers Science Park Harlow UK; 2 Department of Cellular Genomics GlaxoSmithKline Research and Development Gunnels Wood Road Stevenage UK; 3 Paradigm Therapeutics (S) Pte Ltd 10 Biopolis Road #03-01 Chromos Singapore

## Abstract

The aryl hydrocarbon receptor nuclear translocator (*ARNT*) and cathepsin K (*CTSK*) genes lie in a tandem head-to-tail arrangement on human chromosome 1. The two genes are in extremely close proximity; the usual *CTSK* transcription start site is less than 1.4 kb downstream of the end of the longest reported *ARNT* transcript. By generating an RT-PCR product that overlaps both the 3′ end of *ARNT* and the 5′ end of *CTSK*, we show that *ARNT* transcripts may extend through the *ARNT*–*CTSK* intergenic region and progress into the *CTSK* gene. Furthermore, by using quantitative RT-PCR from several tissues to detect the *ARNT* expression signature in *CTSK* introns, we show that *ARNT* transcripts can read through into *CTSK* as far as *CTSK* intron 3, extending approximately 3.7 kb downstream of the end of the longest previously described *ARNT* mRNA. Given that *ARNT* and *CTSK* are expressed in an overlapping range of tissues, *ARNT* read-through may have a negative impact on *CTSK* transcript levels by interfering with *CTSK* expression. We also present evidence for novel *CTSK* transcripts following sequence analysis of *CTSK*-derived ESTs and RT-PCR products. These transcripts show alternate 5′ splicing and or 5′ extension and are sometimes initiated from a cryptic alternative promoter which is upstream of the known *CTSK* promoter and possibly in the 3′ UTR of *ARNT*.


**Comparative and Functional Genomics**
*Comp Funct Genom* 2005; **6**: 268–276.
Published online 11 July 2005 in Wiley InterScience (www.interscience.wiley.com). **DOI:** 10.1002/cfg.483

**Research Article**

### Atypical *CTSK* transcripts and *ARNT* transcription read-through into *CTSK*

Fabienne S. Giraudeau[1], Jean-Philippe Walhin[2], Paul R. Murdock[2], Nigel K. Spurr[1] and Ian C. Gray[1#*]
[1] *Department of Discovery Genetics, GlaxoSmithKline Research and Development, New Frontiers Science Park, Harlow, UK*
[2] *Department of Cellular Genomics, GlaxoSmithKline Research and Development, Gunnels Wood Road, Stevenage, UK*

**Correspondence to: Ian C. Gray, Paradigm Therapeutics (S) Pte Ltd, 10 Biopolis Road, #03-01 Chromos, Singapore. E-mail: igray@paradigm-therapeutics.com*

*# Current address: Paradigm Therapeutics (S) Pte Ltd, 10 Biopolis Road, #03-01 Chromos, Singapore.*

### Abstract

**The aryl hydrocarbon receptor nuclear translocator (*ARNT*) and cathepsin K (*CTSK*) genes lie in a tandem head-to-tail arrangement on human chromosome 1. The two genes are in extremely close proximity; the usual *CTSK* transcription start site is less than 1.4 kb downstream of the end of the longest reported *ARNT* transcript. By generating an RT-PCR product that overlaps both the 3′ end of *ARNT* and the 5′ end of *CTSK*, we show that *ARNT* transcripts may extend through the *ARNT–CTSK* intergenic region and progress into the *CTSK* gene. Furthermore, by using quantitative RT-PCR from several tissues to detect the *ARNT* expression signature in *CTSK* introns, we show that *ARNT* transcripts can read through into *CTSK* as far as *CTSK* intron 3, extending approximately 3.7 kb downstream of the end of the longest previously described *ARNT* mRNA. Given that *ARNT* and *CTSK* are expressed in an overlapping range of tissues, *ARNT* read-through may have a negative impact on *CTSK* transcript levels by interfering with *CTSK* expression. We also present evidence for novel *CTSK* transcripts following sequence analysis of *CTSK*-derived ESTs and RT-PCR products. These transcripts show alternate 5′ splicing and or 5′ extension and are sometimes initiated from a cryptic alternative promoter which is upstream of the known *CTSK* promoter and possibly in the 3′ UTR of *ARNT*. Copyright © 2005 John Wiley & Sons, Ltd.**

**Keywords:** cathepsin k; *CTSK*; aryl hydrocarbon receptor nuclear translocator; *ARNT*; novel transcripts; transcription overlap

**Supplementary material for this article can be found at: http://www.interscience.wiley.com/jpages/1531-6912/suppmat/**

Received: 7 November 2004
Revised: 18 April 2005
Accepted: 9 May 2005

### Introduction

Cathepsin K (CTSK) is a cysteine protease of the papain family. Substrates include type I collagen and osteonectin, major components of the organic matrix of bone (Bossard *et al.*, 1996; Bromme *et al.*, 1996) suggesting that CTSK plays a critical role in osteoclast-mediated bone degradation. Although the predominant site of expression appears to be the osteoclast, consistent with a role for CTSK in bone remodelling, CTSK transcripts have also been detected in a range of other tissues (Bromme and Okamoto, 1995; Inaoka *et al.*, 1995; Drake *et al.*, 1996). The gene encoding CTSK consists of eight exons spread over approximately 12 kb on chromosome 1 (Rood *et al.*, 1997). The structure of the gene is unremarkable, but it is in very close proximity to the neighbouring upstream gene, the aryl hydrocarbon receptor nuclear translocator (*ARNT*) gene; the accepted *CTSK* transcription start site is less than 1.4 kb downstream of the end of the *ARNT* transcript reference sequence (UCSC genome browser; **http://genome.ucsc.edu/**). The *ARNT* gene has 22 exons spanning 65 kb (Scheel and Schrenk, 2000). The gene product, ARNT, is a basic helix–loop–helix protein that complexes with the

Copyright © 2005 John Wiley & Sons, Ltd.

aryl hydrocarbon receptor (AHR) or one of a number of other protein partners to form transcription factors involved in the activation of genes in several biological pathways, including xenobiotic response, neurogenesis, angiogenesis and response to hypoxia (Swanson, 2002). *ARNT* is expressed in a wide range of tissues (Carter *et al.*, 1994), many of which also express CTSK.

Given the extremely close proximity of the two genes, highly efficient termination of *ARNT* transcription would be required to prevent occasional read-through into *CTSK*. Although described for yeast (Greger and Proudfoot, 1998), where a compact genome places many genes close to their neighbours, observation of transcription read-through into an adjacent downstream gene in a mammalian genome is extremely rare. Transcription read-through has implications for the regulation of gene expression; studies using plasmid constructs in cell-based assay systems have shown that read-through can interfere with expression of the downstream gene, resulting in reduced transcript levels (Proudfoot, 1986; Henderson *et al.*, 1989; Greger and Proudfoot, 1998). Using RT-PCR, we show that *ARNT* transcripts can indeed read through into *CTSK*, extending beyond *CTSK* exon 3. We also present evidence for novel *CTSK* transcripts, some of which are initiated from a cryptic alternative promoter, possibly residing in the 3′ UTR of *ARNT*.

### Methods

### EST identification

ESTs were identified by querying the GenBank human EST database with *CTSK* introns and the *ARNT–CTSK* intergenic region, using the BLAST (Altschul *et al.*, 1990) server hosted at the National Center for Biotechnology Information (NCBI:**http://www.ncbi.nlm.nih.gov/BLAST**), using the default software settings.

### DNA sequencing

Following template preparation using standard methods, EST clones (AA361613, BI826433 and BM722737) and PCR products were sequenced using a Big Dye Terminator Kit (Applied Biosystems, Foster City, CA) and an Applied Biosystems 3700 or 3100 PRISM sequencing system in accordance with the manufacturer's instructions.

### cDNA preparation cDNA was prepared as described in Chapman *et al.* (2000). Briefly, human tissue or RNA was purchased (Biochain, San Leandro, CA; Invitrogen, Leek, The Netherlands; Clontech, Palo Alto, CA) or donated (Netherlands Brain Bank, Amsterdam, The Netherlands) and poly $A^+$ RNA prepared from 10 tissue types (brain, heart, lung, liver, kidney, skeletal muscle, intestine, spleen/lymphocyte, placenta and testis) by the PolyATract method (Promega, Madison, WI) in accordance with the kit supplier's instructions. Poly $A^+$ RNA samples were quantitated using $OD_{260nm}$ measurement or the RiboGreen fluorescent method (Molecular Probes, Eugene, OR) and 1 μg each RNA was reverse-transcribed using random nonamers and Superscript II reverse transcriptase (Invitrogen, San Diego, CA) according to the supplier's instructions. The cDNA prepared was diluted and arrayed to produce replica 96-well plates using a Biomek robot (Beckman Coulter, High Wycombe, UK), such that each of the wells contained the cDNA produced from 1 ng RNA for the appropriate tissue. Plates were stored at −80 °C prior to use.

### SYBR Green RT-PCR

SYBR green quantitative RT-PCR (Applied Biosystems) was performed in accordance with kit supplier's guidelines, using cDNA templates prepared as above. PCR reactions were performed in 1× SYBR green PCR master mix with 400 nM each gene-specific primer (Table 1) and 1 ng template. Following initial incubations at 50 °C for 2 min and 95 °C for 10 min, samples were subjected to 40 cycles of PCR (95 °C for 1 min, 50 °C for 1 min, 59 °C for 1 min), using an ABI PRISM 7700 thermal cycler and signal detection instrument. In addition to the sequences under study, three housekeeping genes were measured ($\beta$-actin, GAPDH and cyclophilin) to check RNA quality and quantity.

### PCR from cDNA

cDNA samples from five different tissues, heart, skeletal muscle, macrophage, adipose and bone,

Copyright © 2005 John Wiley & Sons, Ltd. 

**Table 1.** PCR primers

| Region | Primer pair | |
|---|---|---|
| *ARNT* 3′ UTR | TCTTGATTGCGGCTTTATCATTC | TGGAGCTTAAACTATAGATTCCTCTGG |
| Intergenic (ESTAA361613) | AGTTGTCATTACTTCCAGGCAGAAT | TCAGCAAAACCACATTAGGCTGGGAAAA |
| *CTSK* exon 2 | GCTCAAGGTTCTGCTGCTACCT | CTCCCAGTGGGTGTCCAGTATC |
| *CTSK* intron 1 | AGTCCTTGGAACCAGATGTACCA | TGAGGAAGAGAAAGGTAGACGGA |
| *CTSK* intron 3 | GCAAGTATAGCTTCAGCTCCTGTC | AGGGAACTAAAGCAAATGGTGC |
| *CTSK* intron 4 | CACATAGCTACTGGGTGGCAAAG | GGCATTTGTCCTGGGAGCTA |
| *CTSK* intron 6 | TGCAGTATGGAGCAGCATCTCT | CCCAGTCTAGGAGACTGTTTGAAGA |
| *CTSK* intron 7 | CCCAGGTCCAAGCACTTACC | TCCTCCCAGCTCTCTTGGAA |
| $\beta$-Actin | GAGCTACGAGCTGCCTGACG | GTAGTTTCGTGGATGCCACAGGA |
| *GAPDH* | CAAGGTCATCCATGACAACTTTG | ACCACAGTCCATGCCATCACTGCCA |
| Cyclophilin | CATCTGCACTGCCAAGACTGA | CCAAACACCACATGCTTGCCATCCA |
| Intragenic → exon 2 | TCTGGGCATATCCTCCTTCA | GTGGGTGTCCAGTATCTCCT |
| Intron 1 → exon 3 | AGAATGAGGAGATATACAATGT | CCCCCAGGTGGTTCATAGC |
| *ARNT* 3′ UTR → *CTSK* exon 1 | GAGAGAGGGGAAGAGTCGGGA | CTGCTGATGGAAATCTGTTGTCT |

The first 11 primer pairs listed were used for SYBR green quantitative PCR. The three remaining pairs were used for amplification of novel *CTSK* 5′ splice variants ('intragenic → exon 2' and 'intron 1 → exon 3') and an *ARNT–CTSK* overlapping transcript ('*ARNT* 3′ UTR → *CTSK* exon 1') from cDNA.

prepared as described above, were used. 40 pg polyA cDNA were amplified in a total volume of 20 µl with a concentration of 1 µM for each primer, 1 mM dNTPs, 2 µl 10× buffer (Qiagen, The Netherlands), 4 µl buffer Q (Qiagen) and 0.5 U Hot-start Taq Polymerase (Qiagen). PCR was performed using a Peltier thermal cycler (Model PTC-225) using the following parameters: a denaturing step at 94 °C for 10 min, followed by 40 cycles as follows: 94 °C for 1 min, an annealing step at 58.4 °C for 1 min, 72 °C for 3 min, followed by a final extension at 72 °C for 10 min. Primer sequences are given in Table 1. PCR products were separated by agarose gel electrophoresis and purified using a gel extraction kit (Qiagen) prior to sequencing as described above.

### Test for correlation between expression levels

The Pearson correlation coefficient for each pairwise comparison of expression levels for different genomic regions was calculated using the CORREL function in Microsoft Excel. The test statistic ($t$) was calculated using the formula:

$$t = r\sqrt{\frac{n-2}{1-r^2}}$$

where $r$ = correlation coefficient and $n$ = number of tissues analysed. $p$-Values were calculated using the TDIST function in Microsoft Excel (one-tailed test).

### Results

### Novel *CTSK* 5′ splicing and 5′ extended *CTSK* transcripts

The UCSC 'Golden Path' Genome Browser (**http://genome.ucsc.edu/**) places the transcription start site of the *CTSK* gene, as defined by the *CTSK* reference sequence NM000396 (Li *et al.*, 2004), less than 1.4 kb downstream of the 3′ end of the *ARNT* gene, as defined by the *ARNT* reference transcript NM_001668 (Chapman-Smith *et al.*, 2004). This is similar to the arrangement in the mouse genome, where a 4.5 kb stretch of DNA separates the *Arnt* and *Ctsk* transcripts (Rantakokko *et al.*, 1999). A BLAST search of the NCBI human EST database (**http://www.ncbi.nlm.nih.gov/BLAST/**), using the *ARNT–CTSK* intergenic sequence as a query sequence, returned numerous ESTs with perfect or near-perfect matches. We also identified several ESTs in *CTSK* introns 1, 2 and 3, some of which bridge the exon–intron boundaries, suggesting that these intergenic and intronic regions show significant levels of expression in a number of tissue types (Figure 1). By contrast, very few ESTs were retrieved by querying the database with the remaining five *CTSK* introns (other than those matching Alu repeats in introns 4 and 5 and a MER repeat in intron 7, not shown).

In order to characterize the putative intergenic and *CTSK* 5′ intron transcripts further, three ESTs were selected for further sequencing, two

Copyright © 2005 John Wiley & Sons, Ltd. 

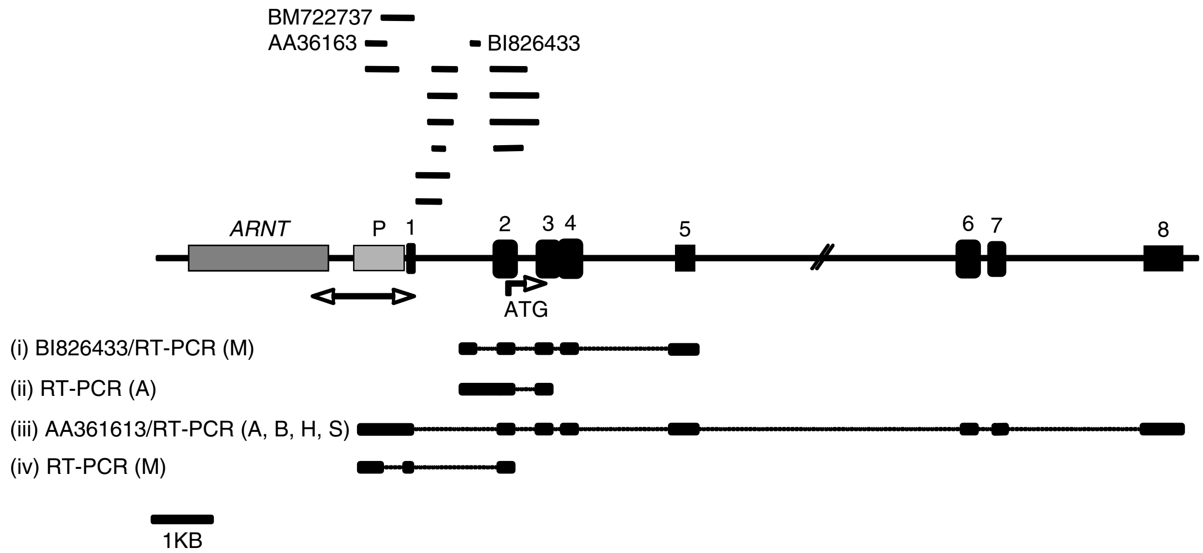

**Figure 1.** The *CTSK* gene and associated novel transcripts. Exons are represented by black bars and are numbered. The position of the translation start site in exon 2 is shown by an arrow labelled ATG. The palegrey box immediately upstream of exon 1 marked P represents the promoter described by Motyckova *et al.* (2001). The grey box upstream of *CTSK* marked *ARNT* represents the final exon of the *ARNT* gene. ESTs identified from querying the GenBank human EST database are depicted as black lines above the 5′ end of the *CTSK* gene; those selected for further sequencing are named AA36163, BM22737 and BI826433. Novel cDNA sequences identified by EST sequencing and RT-PCR are shown below the *CTSK* gene; solid black lines represent mRNA and faint dotted lines correspond to regions that are spliced out. Splice form (i) corresponds to clone BI826433; the novel splicing pattern at the 5′ end of this variant was confirmed by PCR from macrophage (M) cDNA. Splice form (ii) was identified by PCR from adipose (A) cDNA. Splice form (iii) corresponds to clones AA361613 and BM722737 and was confirmed by PCR from adipose (A), bone (B), heart (H) and skeletal muscle (S) cDNA. Splice form (iv) was identified by PCR from macrophage (M) cDNA. Although not demonstrated empirically, it seems likely that splice forms (i), (ii) and (iv), like splice form (iii), can form part of full-length *CTSK* transcripts. The line with an arrowhead at each end, below the *ARNT–CTSK* intergenic region, shows the location of an RT-PCR product representing an *ARNT–CTSK* overlapping transcript generated from heart, skeletal muscle, adipose and bone tissue. The sequences of novel splice forms (i)–(iv) are shown in Supplementary Figure 1

from the intergenic *ARNT–CTSK* region (GenBank AA361613 and BM722737, derived from T-lymphocyte and fetal eye, respectively) and one from *CTSK* intron 1 (GenBank BI826433, derived from medulla). Sequence analysis of the clones represented by these ESTs revealed that clone BI826433 is a partial *CTSK* cDNA extending from what appears to be an alternative first exon located within intron 1. The novel exon 1 is correctly spliced to exon 2 and the clone extends as far as exon 5, with exons 2–5 correctly spliced (Figure 1). Using primers extending from EST BI826433 to *CTSK* exon 3, we were able to PCR amplify this splice form from macrophage cDNA, providing evidence that this is a genuine transcript rather than an artefact (Figure 2A). We identified a second alternative splice form in cDNA from adipose tissue, extending unspliced from within intron 1 to exon 2, but with exon 2 correctly spliced to exon 3 (Figures 1, 2A). As the *CTSK* ATG translation start signal is in exon 2, it is possible for transcripts that are missing exon 1 or have an alternative first exon to encode a complete CTSK protein with the correct amino acid sequence.

ESTs AA361613 and BM722737, which map to the *ARNT–CTSK* intergenic region, both represent full-length conventionally spliced *CTSK* cDNA clones which extend the 5′ end of the *CTSK* transcript relative to the *CTSK* reference sequence NM000396 (Figure 1); clone AA36163, the longer of the two, extends the *CTSK* mRNA by 722 bases. As with clone BI826433, we were able to confirm that these clones represent genuine transcripts by PCR amplification of 5′ extended transcripts with correct intron 1 splicing from cDNA samples from a range of tissues (Figure 2B). We observed an alternative transcript

Copyright © 2005 John Wiley & Sons, Ltd.

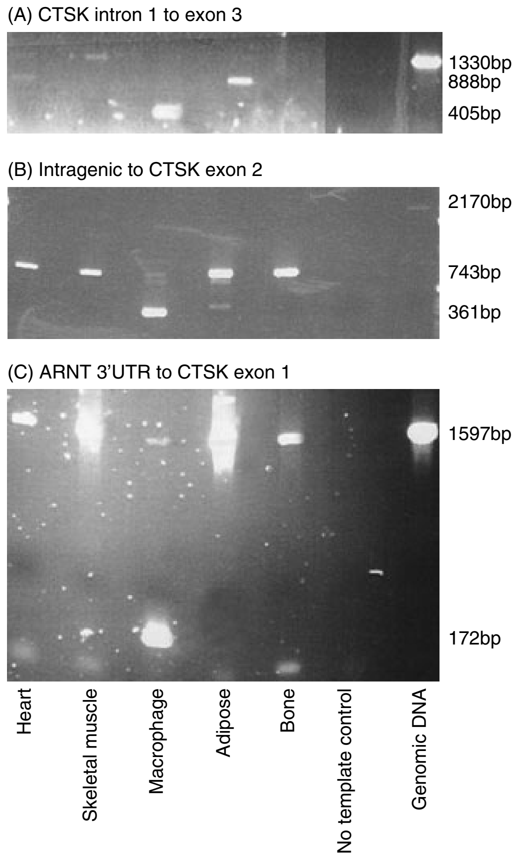

**Figure 2.** RT-PCR products from the *ARNT–CTSK* locus (see Table 1 for primer sequences). (A) RT-PCR products amplified using primers from *CTSK* intron 1 and exon 3. The 405 bp and 888 bp fragments from macrophage and adipose respectively represent the 5′ ends of novel splice forms (i) and (ii) shown in Figure 1. The 888 bp product also appears to be present in heart, but the band is faint. No products were generated from bone and only a faint unspliced product was generated from skeletal muscle. The 1330 bp unspliced genomic DNA product is shown for comparison. (B) RT-PCR products amplified using primers from the *ARNT-CTSK* intergenic region and *CTSK* exon 2. All tissues tested, with the exception of macrophage, yield a 743 bp fragment corresponding to the 5′ end of novel splice form (iii) shown in Figure 1. Macrophage cDNA gives a 361 bp fragment representing novel splice form (iv) shown in Figure 1. The 2170 bp unspliced genomic DNA product is shown for comparison (but is faint). (C) RT-PCR products amplified using primers from *ARNT* 3′ UTR and *CTSK* exon 1. All tissues tested give an unspliced 1597 bp product consistent with read-through from *ARNT* into *CTSK*. However, the predominant species from macrophage is a 172 bp product where the entire intergenic region has been spliced out, joining the *ARNT* 3 UTR to *CTSK* exon 1. The sequence of this product is shown in Supplementary Figure 2. (The very small product faintly visible in all lanes, but most obvious in bone, is thought to be a primer dimer)

in macrophage-derived cDNA, with a novel intron upstream of exon 1 spliced out (Figures 1, 2B). Significantly, these 5′ extended transcripts stretch back across almost the entire length of the 903 bp *CTSK* promoter characterized by Motyckova *et al.* (2001) and are presumably driven from an alternative cryptic promoter element further upstream.

### *ARNT* read-through into *CTSK*

To gauge the relative expression level of the 5′ extended *CTSK* message, we compared SYBR green quantitative RT-PCR profiles (Wittwer *et al.*, 1997) from the 5′ extended ('intergenic') region and *CTSK* exon 2 in a number of tissue types. *ARNT* expression levels were also measured by including the 3′ UTR of *ARNT* in this analysis. Expression of the intergenic region was detected in all tissues tested, at widely varying levels (Figure 3A).

Although there is some evidence for correlation between the *CTSK* expression pattern and the expression profile of the intergenic region (the correlation coefficient, $r^2$, is 0.38, giving a $p$-value of 0.03; Table 2), the correlation between the intergenic region and *ARNT* expression patterns is far stronger ($r^2 = 0.82$, $p = 1 \times 10^{-4}$; Table 2). This led us to hypothesize that this region can form part of either *CTSK* or *ARNT* messages, due to *ARNT* read-through into the intergenic region in addition to 5′ extension of *CTSK* transcripts. It should be noted there is no significant correlation between the conventional *CTSK* and *ARNT* expression patterns ($r^2 = 0.23$, $p = 0.08$; Table 2). Transcript overlap, most likely to be due predominantly to *ARNT* read-through into *CTSK,* was confirmed by amplification of a 1.6 kb fragment extending from the 3′ UTR of *ARNT* to *CTSK* exon 1 from cDNA samples derived from heart, skeletal muscle, macrophage, adipose tissue and bone (Figures 1, 2C). The identity of this fragment was confirmed by sequencing. Prior to reverse transcription, mRNA samples were tested for the presence of genomic DNA by PCR (not shown); no evidence for contaminating DNA was found, supporting the supposition that this unspliced fragment is derived from cDNA and is not residual genomic DNA carried over into the cDNA samples. Furthermore, we identified a 172 bp fragment from macrophage cDNA (Figure 2C), where the entire intergenic region had been spliced

Copyright © 2005 John Wiley & Sons, Ltd.

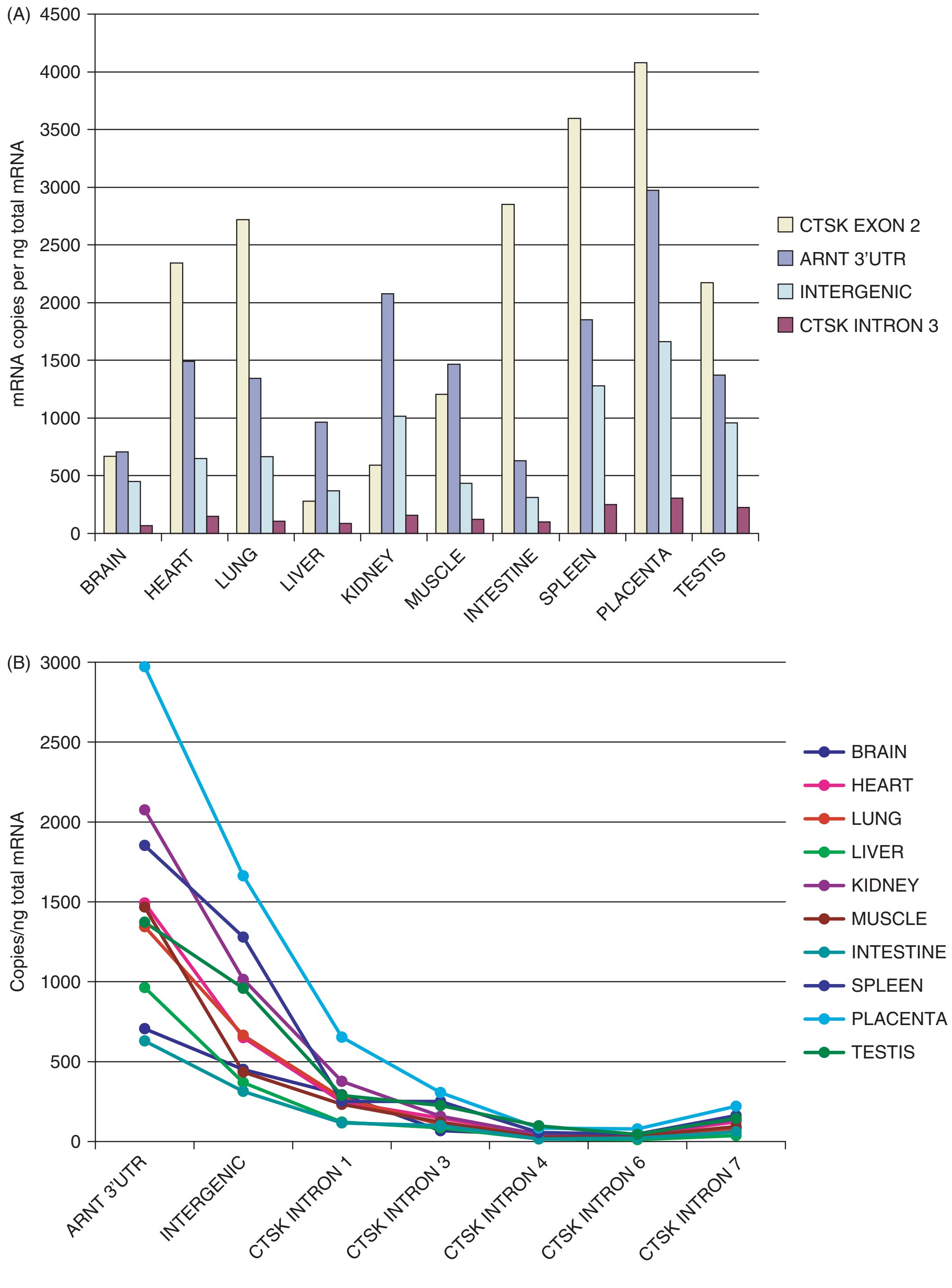

**Figure 3.** Intergenic and intronic expression profiles indicating *ARNT* read-through into *CTSK*. (A) SYBR green quantitative PCR expression profiles for *ARNT* 3′ UTR, *CTSK* exon 2, the intergenic region and *CTSK* intron 3 from a range of tissues, showing that the intergenic region and *CTSK* intron 3 are transcribed and that their transcription profiles have more similarity to *ARNT* than to *CTSK*. Correlation of *CTSK* intron 3 (or the intergenic region) with *ARNT* is far higher than with *CTSK* exon 2 (see Table 2), providing evidence for *ARNT* read-through into *CTSK*. (B) Expression levels at ostensible non-coding regions of the *CTSK* locus. From the *ARNT* 3′ UTR to *CTSK* intron 4 a steady decline in *ARNT*-like expression is apparent. Expression levels for the 3′ *CTSK* introns 4, 6 and 7 are negligible

Copyright © 2005 John Wiley & Sons, Ltd.

**Table 2.** Correlation between expression profiles for *ARNT*, the intergenic region, *CTSK* exon 2 and *CTSK* intron 3

| | *ARNT* 3′ UTR | Intergenic | *CTSK* exon 2 | *CTSK* intron 3 |
|---|---|---|---|---|
| *ARNT* 3′ UTR | — | $r^2 = 0.82$ $p = 1 \times 10^{-4}$ | $r^2 = 0.23$ $p = 0.08$ | $r^2 = 0.70$ $p = 0.001$ |
| Intergenic | — | — | $r^2 = 0.38$ $p = 0.03$ | $r^2 = 0.89$ $p = 2.4 \times 10^{-5}$ |
| *CTSK* exon 2 | — | — | — | $r^2 = 0.49$ $p = 0.01$ |
| *CTSK* intron 3 | — | — | — | — |

Transcript copy numbering total RNA was assessed by SYBR green quantitative RT-PCR for each locus and pairwise comparisons of expression levels made across 10 tissues (brain, heart, lung, liver, kidney, muscle, intestine, spleen, placenta and testis). Corresponding scatter plots are shown in Supplementary Figure S2. $r^2$ = the square of the Pearson correlation coefficient. $p$-Values were calculated using a one-tailed $t$-test.

out, fusing the *ARNT* 3′ UTR with *CTSK* exon 1, using the same splice acceptor as the novel macrophage transcript (iv) shown in Figure 1.

In order to determine the extent of any potential *ARNT* read-through, we analysed *CTSK* introns 1, 3, 4, 6 and 7 and found evidence for transcription. Although much lower than *ARNT* 3′ UTR expression levels, profiles derived from the *CTSK* introns correlate strongly with those for *ARNT*, supporting the view that these transcripts represent *ARNT* transcription read-through into *CTSK* (Table 2, Figure 3). The expression profiles from *CTSK* introns are highly correlated with expression of both the intergenic region and *ARNT*. For example, correlation of *CTSK* intron 3 expression with expression of the intergenic region gives an $r^2$ value of 0.89 and a $p$ value of $2.4 \times 10^{-5}$ (Table 2). Similarly, $r^2 = 0.70$, $p = 0.001$ when comparing *CTSK* intron 3 expression with *ARNT* expression (Table 2). There is also some evidence for weak correlation between the expression profiles of *CTSK* exon 2 and intron 3 ($r^2 = 0.49$, $p = 0.01$; Table 2), possibly due to the presence of small amounts of unspliced, alternately spliced or partially spliced RNA in the mRNA samples. To ensure that the *ARNT* 3′ UTR was giving a true representation of *ARNT* gene expression, we also measured expression levels of *ARNT* coding sequence, with the same outcome (not shown). The *ARNT*-like expression profile declines steadily through introns 1–3 and becomes negligible in introns 4, 6 and 7 (Figure 3C), suggesting that read-through can extend as far as *CTSK* intron 3, approximately 3.7 kb downstream of the end of the longest previously described *ARNT* mRNA (Chapman-Smith *et al.*, 2004).

### Discussion

The significance of the alternative *CTSK* transcripts and 5′ splice forms is unclear. CTSK is translated as an inactive precursor (preprocathepsin K) comprising a 15 amino acid N-terminal secretion signal sequence followed by a 99 amino acid leader sequence; the active form of CTSK is formed by cleavage and removal of the leader sequence (Bromme *et al.*, 1996, Bossard *et al.*, 1996). Therefore, although splice forms 1 and 2 (Figure 1) could potentially add further amino acids to the N-terminus of CTSK, due to the presence of upstream in-frame ATG codons (not shown), the production of novel protein isoforms from these splice variants seems unlikely. Furthermore, no higher molecular weight species corresponding to proteins of increased length have been observed by Western blotting (Rieman *et al.*, 2001; Hou *et al.*, 2002).

Although CTSK may be unaffected at the protein level, the alternative 5′ ends may confer increased or decreased message stability compared to the conventional splicing pattern. Alternatively these splice forms may be of little functional consequence and simply contribute a minor additional component to overall *CTSK* transcript levels. The longer transcripts (splice forms 3 and 4) appear to be transcribed from an alternative promoter in either the intergenic region immediately upstream of the known *CTSK* promoter or possibly in the 3′ UTR of *ARNT*. Given the poor efficiency of this cryptic promoter (reflected in the low levels of *CTSK* expression driven from it), it may not be a *bona fide* promoter *per se*, but rather a sequence from which 'leaky' expression occurs due to low-level recruitment of transcription factors.

The *ARNT* cDNA reference sequence NM_001668 (Chapman-Smith *et al.*, 2004) has 2.3 kb of 3′ UTR extending to within 1.4 kb of the transcription start site of *CTSK* (see UCSC human genome browser). A large number of ESTs mapping across the entire 2.3 kb of *ARNT* 3′

Copyright © 2005 John Wiley & Sons, Ltd. 

UTR attest to the conclusion that the *ARNT* transcript frequently reaches such a length. However, shorter *ARNT* transcripts have also been reported, with 3′ UTR in the range 178–1929 bp (GenBank references BC028362, AL834279 and M69238), implying that termination of *ARNT* transcription can occur at any one of a number of places downstream of the coding sequence. Putative polyadenylation signals (AATAAA) lie 78 bp and 726 bp downstream of the TAG termination codon; these signals may be employed to generate the shorter splice forms, although no T-rich downstream sequence element (DSE), which is also required for efficient polyadenylation (Chen *et al.*, 1995), is apparent for either. Transcription termination is dependent on a functional polyadenylation signal (see Proudfoot, 2002) and the existence of transcripts with extensive 3′ UTR suggests that the *ARNT* AATAAA motifs act as weak signals, often failing to trigger termination and allowing transcript formation to continue. No consensus polyadenylation signals are apparent at the 3′ end of the longer transcripts, suggesting that termination of transcription is likely to be highly inefficient for these already extended messages, leading to transcription read-through into the adjacent *CTSK* gene. We cannot exclude the formal possibility that the 1.6 kb transcript fragment that we identified, extending from the 3′ UTR of *ARNT* to *CTSK* exon 1, together with the 172 bp spliced form identified from macrophage cDNA, represent 5′ extended *CTSK* transcripts driven from a cryptic promoter within the *ARNT* gene. However, the identification of the *ARNT* expression signature in the intergenic region and in 5′ *CTSK* introns strongly suggests that transcription read-through from *ARNT* into *CTSK* is the more likely explanation.

There are other examples of overlapping transcripts in the human and mouse genomes, although the majority described to date are overlaps at the 3′ end of genes in tail-to-tail orientation (see e.g. Williams and Fried, 1986, Campbell *et al.*, 1997; Dear *et al.*, 2000). Several examples of overlapping gene groups, where exons of one gene are contained within the introns of another, have also been described (Karlin *et al.*, 2002). However, to our knowledge examples of transcription read-through as described in this paper have not been reported previously for mammalian genomes. Like *ARNT* and *CTSK*, the Rab geranylgeranyl transferase $\alpha$-subunit gene (*RABGGTA*) and transglutaminase 1 gene (*TGM1*) are arranged in tandem orientation with less than 2 kb separating the 3′ end of *RABGGTA* from the transcription initiation site of *TGM1*. Putative regulatory elements that influence *TGM1* expression are found in the 3′ end of the *RABGGTA* transcribed region (Van Bokhoven *et al.*, 1996), although at present there is no evidence for *RABGGTA* transcription read-through into the *TGM1* coding sequence.

Studies with yeast and cell-based mammalian assay systems have shown that transcription read-through can reduce expression of the adjacent downstream gene (Proudfoot, 1986; Henderson *et al.*, 1989; Greger and Proudfoot, 1998). Given that *ARNT* and *CTSK* are co-expressed in a number of tissues (Figure 3), it is possible that *ARNT* read-through has a negative impact on *CTSK* expression. Although rarely reported to date, transcription read-through into a nearby gene, particularly at low levels, may be a fairly common phenomenon in the mammalian genome and, as postulated several years ago (Proudfoot, 1986), could be an important factor in the regulation of mammalian gene expression. Functional studies will be required to assess the molecular and physiological impact of *ARNT* read-through into *CTSK*.

### Acknowledgements

The authors would like to thank Dr R. Ravid (Netherlands Brain Bank, The Netherlands) for arrangement/donation of human brain tissue.

### References

Altschul SF, Gish W, Miller W, Myers EW, Lipman DJ. 1990. Basic local alignment search tool. *J Mol Biol* **215**: 403–410.

Bossard MJ, Tomaszek TA, Thompson SK, *et al.* 1996. Proteolytic activity of human osteoclast cathepsin K. Expression, purification, activation, and substrate identification. *J Biol Chem* **271**: 12 517–12 524.

Bromme D, Okamoto K. 1995. Human cathepsin O2, a novel cysteine protease highly expressed in osteoclastomas and ovary: molecular cloning, sequencing and tissue distribution. *Biol Chem Hoppe Seyler* **376**: 379–384.

Bromme D, Okamoto K, Wang BB, Biroc S. 1996. Human cathepsin O2, a matrix protein-degrading cysteine protease expressed in osteoclasts. Functional expression of human cathepsin O2 in *Spodoptera frugiperda* and characterization of the enzyme. *J Biol Chem* **271**: 2126–2132.

Campbell HD, Fountain S, Young IG, *et al.* 1997. Genomic structure, evolution, and expression of human FLII, a gelsolin and leucine-rich-repeat family member: overlap with LLGL. *Genomics* **42**: 46–54.

Copyright © 2005 John Wiley & Sons, Ltd. 

Carver LA, Hogenesch JB, Bradfield CA. 1994. Tissue specific expression of the rat Ah-receptor and ARNT mRNAs. *Nucleic Acids Res* **22**: 3038–3044.

Chapman CG, Meadows HJ, Godden RJ, *et al.* 2000. Cloning, localisation and functional expression of a novel human, cerebellum specific, two pore domain potassium channel. *Brain Res Mol Brain Res* **82**: 74–83.

Chapman-Smith A, Lutwyche JK, Whitelaw ML. 2004. Contribution of the Per/Arnt/Sim (PAS) domains to DNA binding by the basic helix–loop–helix PAS transcriptional regulators. *J Biol Chem* **279**: 5353–5362.

Chen F, MacDonald CC, Wilusz J. 1995. Cleavage site determinants in the mammalian polyadenylation signal. *Nucleic Acids Res* **23**: 2614–2620.

Dear TN, Meier NT, Hunn M, Boehm T. 2000. Gene structure, chromosomal localization, and expression pattern of Capn12, a new member of the calpain large subunit gene family. *Genomics* **68**: 152–160.

Drake FH, Dodds RA, James IE, *et al.* 1996. Cathepsin K, but not cathepsins B, L, or S, is abundantly expressed in human osteoclasts. *J Biol Chem* **271**: 12 511–12 516.

Greger IH, Proudfoot N. 1998. Poly(A) signals control both transcriptional termination and initiation between the tandem GAL10 and GAL7 genes of *Saccharomyces cerevisiae*. *EMBO J* **17**: 4771–4779.

Henderson SL, Ryan K, Sollner-Webb B. 1989. The promoter-proximal rDNA terminator augments initiation by preventing disruption of the stable transcription complex caused by polymerase read-in. *Genes Dev* **3**: 212–223.

Hou WS, Li W, Keyszer G, *et al.* 2002. Comparison of cathepsins K and S expression within the rheumatoid and osteoarthritic synovium. *Arthrit Rheum* **46**: 663–674.

Inaoka T, Bilbe G, Ishibashi O, *et al.* 1995. Molecular cloning of human cDNA for cathepsin K: novel cysteine proteinase predominantly expressed in bone. *Biochem Biophys Res Commun* **206**: 89–96.

Karlin S, Chen C, Gentles AJ, Cleary M. 2002. Associations between human disease genes and overlapping gene groups and multiple amino acid runs. *Proc Natl Acad Sci USA* **99**: 17 008–17 013.

Li Z, Yasuda Y, Li W, *et al.* 2004. Regulation of collagenase activities of human cathepsins by glycosaminoglycans. *J Biol Chem* **279**: 5470–5479.

Motyckova G, Weilbaecher KN, Horstmann M, *et al.* 2001. Linking osteopetrosis and pycnodysostosis: regulation of cathepsin K expression by the microphthalmia transcription factor family. *Proc Natl Acad Sci USA* **98**: 5798–5803.

National Center for Biotechnology Information BLAST server: **http://www.ncbi.nlm.nih.gov/BLAST**.

Proudfoot NJ. 1986. Transcriptional interference and termination between duplicated $\alpha$-globin gene constructs suggests a novel mechanism for gene regulation. *Nature* **322**: 562–565.

Proudfoot NJ, Furger A, Dye MJ. 2002. Integrating mRNA processing with transcription. *Cell* **108**: 501–512.

Rantakokko J, Kiviranta R, Eerola R, Aro HT, Vuorio E. 1999. Complete genomic structure of the mouse cathepsin K gene (Ctsk) and its localization next to the Arnt gene on mouse chromosome 3. *Matrix Biol* **18**: 155–61.

Rieman DJ, McClung HA, Dodds RA, *et al.* 2001. Biosynthesis and processing of cathepsin K in cultured human osteoclasts. *Bone* **28**: 282–289.

Rood JA, Van Horn S, Drake FH, Gowen M, Debouck C. 1997. Genomic organization and chromosome localization of the human cathepsin K gene (CTSK). *Genomics* **41**: 169–176.

Scheel J, Schrenk D. 2000. Genomic structure of the human Ah receptor nuclear translocator gene (hARNT). *Hum Genet* **107**: 397–399.

Swanson HI. 2002. DNA binding and protein interactions of the AHR/ARNT heterodimer that facilitate gene activation. *Chem Biol Interact* **141**: 63–76.

University of California, Santa Cruz human genome browser: **http://genome.ucsc.edu/**.

van Bokhoven H, Rawson RB, Merkx GF, Cremers FP, Seabra MC. 1996. cDNA cloning and chromosomal localization of the genes encoding the $\alpha$- and $\beta$-subunits of human Rab geranylgeranyl transferase: the 3′ end of the $\alpha$-subunit gene overlaps with the transglutaminase 1 gene promoter. *Genomics* **38**: 133–140.

Williams T, Fried M. 1986. A mouse locus at which transcription from both DNA strands produces mRNAs complementary at their 3′ ends. *Nature* **322**: 275–279.

Wittwer CT, Herrmann MG, Moss AA, Rasmussen RP. 1997. Continuous fluorescence monitoring of rapid cycle DNA amplification. *Biotechniques* **22**: 134–138.

Copyright © 2005 John Wiley & Sons, Ltd. 
